# ﻿A taxonomic review of *Colobopsis minus* (Wang & Wu, 1994), comb. nov. from China, with description of all castes (Hymenoptera, Formicidae)

**DOI:** 10.3897/zookeys.1260.166957

**Published:** 2025-11-14

**Authors:** Yonghao Ye, Hao Ran, Qionghua Gao

**Affiliations:** 1 Guangxi Key Laboratory of Agro-Environmental and Agric-Products Safety/National Demonstration Center for Experimental Plant Science Education, College of Agriculture, Guangxi University, Nanning, 530004, China Guangxi University Nanning China; 2 State Key Laboratory of Genetic Resources and Evolution, Kunming Institute of Zoology, Chinese Academy of Sciences, Kunming, 650223, China State Key Laboratory of Genetic Resources and Evolution, Kunming Institute of Zoology, Chinese Academy of Sciences Kunming China

**Keywords:** *
Camponotus
*, *
Colobopsis
*, identification key, morphology, phylogeny, taxonomy

## Abstract

*Camponotus* and *Colobopsis* are two species-rich genera within the ant tribe Camponotini. Despite their non-sister phylogenetic relationship, pronounced morphological convergence has long complicated their taxonomic delineation. Here, we clarify the status of *Colobopsis minus* (Wang & Wu, 1994), **comb. nov.**, a taxon historically misassigned to *Camponotus*, through an integrative approach combining COI phylogenetics, caste-specific morphometrics, and pupae type. We provide the first comprehensive description of all castes (minor and major workers, gynes, and males), alongside two taxonomic keys to Chinese *Colobopsis* species. Critical evidence for reclassification includes: (1) phragmotic major workers with *Colobopsis*-specific cephalic modifications; (2) naked pupae and (3) molecular phylogenetic analyses of COI barcode sequences, which robustly place *C. minus* within the *Colobopsis* clade. This study underscores the necessity of synthesizing phylogenetic data with detailed morphological analysis to disentangle complex taxonomic histories in hyperdiverse ant groups.

## ﻿Introduction

*Colobopsis*, a genus in Camponotini of Formicinae (Hymenoptera: Formicidae), was initially established by Mayr (1861). Unlike most C*amponotus*, the major workers of *Colobopsis* usually possess a distinctly truncated (phragmotic) head, with mandibles exhibiting a sharp external ridge, enabling them to block the nest entrance. The head of these species presents a peculiar sculpture consisting of umbilicate punctures, and pupae at all developmental phases lack a cocoon (Wheeler 1904). However, due to the morphological intermediates observed between *Colobopsis* and *Camponotus*, Emery (1889) reclassified *Colobopsis* as a subgenus within *Camponotus*. This taxonomic arrangement persisted through much of the 20^th^ century and was reflected in subsequent works by Emery (1925) and Bolton (2003). However, the taxonomy of *Colobopsis* has been a subject of debate. Phylogenetic studies utilizing multiple nuclear genes and extensive taxon sampling (Brady et al. 2006; Moreau et al. 2006; Moreau and Bell 2013) have suggested that several recognized formicine taxa, including the genus *Camponotus* and tribes Lasiini and Plagiolepidini, are not monophyletic. Molecular studies using UCE (ultra-conserved element) phylogenomic data demonstrated that *Colobopsis* is phylogenetically distinct from *Camponotus*, and is sister to all other genera within Camponotini (Blaimer et al. 2015; Ward et al. 2025). Based on phylogenetic relationships, Ward et al. reinstated *Colobopsis* to full genus level (with 94 species transferred from *Camponotus* to *Colobopsis*), and established diagnostic key to distinguish the two genera using morphological trait (clypeal shape, separation of antennal insertions) and morphometric measurements, enabling myrmecologists to reliably differentiate between them (Ward et al. 2016). Ward and Boudinot (2021) further corroborated this classification through multiple lines of evidence, including worker and male morphology. Their research demonstrated that three species of Camponotus belonging to the subgenus Myrmotemnus are in fact members of the genus *Colobopsis* and seven species previously assigned to *Colobopsis* belong to the genus *Camponotus*. Attempts to develop a straightforward worker-based diagnosis of the two genera have proved difficult due to significant variation within both clades and the complex interplay of convergent and divergent evolution (Ward and Boudinot 2021), highlighting the need for further taxonomic revision.

*Camponotus minus* Wang & Wu, 1994 was originally described as a species closely related to *Camponotus confucii* Forel, date but it can be easily distinguished from the latter by coloration, mesosoma shape, and petiole structure. However, the original diagnosis relied exclusively on textual descriptions and schematic illustrations, with no photographic documentation. Furthermore, the limited type material resulted in incomplete caste descriptions: only the minor worker was definitively described, while the characteristics of major worker and pupae were absent. Consequently, it was long classified within *Camponotus*. However, with the collection of additional samples, the discovery of the phragmotic major worker in *Camponotus minus* and the observation of naked pupae have challenged this classification. Additionally, recent advances in sequencing technology have provided molecular evidence supporting a taxonomic revision.

To resolve these taxonomic uncertainties, we re-examined the holotype specimen and conducted comprehensive sampling across the species’ distribution. Our study provides high-resolution imaging of all castes, quantitative morphometric analyses, and descriptions of all castes. These data facilitated the reassessment of the species’ taxonomic position. Ultimately, by integrating phylogenetic analyses of clades within the subfamily Formicinae with morphological evidence, we formally propose the new combination *Colobopsis minus* (Wang & Wu, 1994), comb. nov.

## ﻿Materials and methods

In this study, specimens of *Colobopsis minus* from two different colonies were used for morphological and molecular analyses. Each colony included workers, gynes, males, and dealate queens, and was collected from Huazhou City, Guangdong Province, China (Suppl. material 1: table S1). Colony GDTQ (Guangdong, Tongqing) was collected on 21 July 2019 from Longdou Village, Tongqing Town (同庆镇龙豆村, 21.63333°N, 110.70000°E), while colony GDGQ (Guangdong, Guanqiao) was collected on 25 May 2020 from Longan Village, Guanqiao Town (官桥镇龙岸村, 21.75000°N, 110.46667°E).

The pinned specimens examined during this study were derived from the colony GDGQ and are preserved in the Insect Collection of Guangxi University (**GXU**), Nanning, Guangxi, China. Morphological observations were made under a Nikon 745T stereomicroscope, and high-quality multi-focused montage images were captured using a Keyence VHX 6000 digital microscope at 200 × magnification. A total of 24 specimens were measured in this study, with detailed data provided in Suppl. material 1: table S2. All measurements are reported in millimeters (mm) and rounded to two decimal places. The morphological terminology follows Longino (2003) and Ward et al. (2016). The following characters were evaluated in this revision:

**ASM** Minimum distance between the antennal sclerites (inter-torular distance).

**CLW** Clypeus width, width of clypeus, taken at the anterior tentorial pits.

**CLL** Clypeus length, maximum measurable length of clypeus, taken along the midline, from a line drawn across posterior margin to a line across the anterior margin (medial indentations on either margin do not decrease length).

**EL** Eye length, length of the compound eye measured along the maximum diameter.

**HL** Head length, midline length of head from the posterior margin to a line across the anterior clypeal margin (medial indentations on either margin do not decrease length).

**HW** Head width, maximum width of head, excluding the eyes.

**PW** Pronotum width, dorsal view, maximum width of the pronotum.

**SL** Scape length, the maximum straight-line length of the scape, excluding the basal constriction or neck that occurs just distal of the condylar bulb.

**TL** Total outstretched length of the ant from the mandibular apex to the gastral apex; when measured in lateral the sum of Mandibular length + head length + mesosomal length + lengths of petiole + length of gaster.

**CI** Cephalic index, HW/HL.

**SI** Scape index, SL/HW.

**REL** Relative eye length, EL/HL.

Genomic DNA was extracted from the flash-frozen workers using Qiagen’s QIAamp DNA Micro Kit (California, USA) according to the manufacturer’s instructions. The mitochondrial COI gene was amplified using the universal primers LCO1490 and HCO2198 for COI (Folmer et al. 1994). PCR products were visualized on agarose gels and subsequently sequenced by Beijing Tsingke Biotechnology. The COI gene sequences generated in this study have been deposited in GenBank (www.ncbi.nlm.nih.gov/genbank) under the accession numbers PV984456 and PV984457 The resulting sequences were aligned with other publicly available Formicinae sequences for phylogenetic analysis (see Suppl. material 1: table S3 for details). Sequence alignment was performed using the ClustalW algorithm implemented in MEGA 11 (Tamura et al. 2021). The best fit nucleotide substitution model, GTR+G+I, was selected based on model selection criteria. Phylogenetic reconstruction was conducted using maximum likelihood (ML) analysis under the GTR+G+I model, with branch support evaluated via bootstrap analysis with 1000 replicates in MEGA 11.

## ﻿Results

### ﻿Taxonomic account

#### ﻿ Colobopsis minus

Taxon classificationAnimaliaHymenopteraFormicidae

(Wang & Wu, 1994) comb. nov.

D7E73926-06B8-511C-820D-00D72DE1D09A

Figs 1, 2, 3, 4, 5

Camponotus minus Wang & Wu, 1994: 26, figs 2, 7, 11 (w.).

##### Type material.

***Holotype*** • worker, China, Guangdong Prov., Dianbai County, 7.iii.1986 (M. Wang) (Natural History Museum of the Chinese Academy of Forestry) [images examined].

##### Additional material.

The newly collected materials of *Colobopsis minus* were obtained by Fengming He from Tongqing Town (同庆镇) and Guanqiao Town (官桥镇), Huazhou City (化州市), which is a county-level city under the administration of Maoming City (茂名市), Guangdong Province. The type locality of *C. minus* is Dianbai County (电白县), now administratively Dianbai District (电白区) of Maoming City. Thus, the new collection sites (Tongqing and Guanqiao Towns, Huazhou City) and the type locality (Dianbai District) all fall within the same broader administrative region (Maoming City, Guangdong Province). The straight-line distance between the new collection sites and the type locality is approximately 50 km. The proximity of these localities, within the same geographic and ecological region, further substantiates the identification of this species.

##### Diagnosis.

Unlike the smooth clypeus of most *Camponotus* species, the anterior one-third of the clypeus in *C. minus*, with the surrounding genae, is abruptly truncate in lateral view. The dorsal sclerite of mesosoma in typical *Camponotus* is generally flat, arched flatter, or bowed; in *C. minus*, the meso-propodeal suture is deeply concave. In lateral view, the base of the propodeum slopes gently from meso-propodeal suture. Basal face basically horizontal, declivity steeply concave, forming a right angle with the base. Additionally, *Camponotus* species often possess a double row of stout spines on the inner surface of the middle and hind tibiae, whereas *C. minus* lacks such elongate setae (Suppl. material 2: fig. S1).

##### Description.

***Major worker*** (*n* = 6): TL 5.33–6.06, HL 1.39–1.62, HW 1.37–1.57, ASM 0.60–0.71, CLL 0.43–0.55, CLW 0.41–0.50, SL 0.89–0.99, EL 0.32–0.35, CI 0.94–0.99, SI 0.60–0.65, PW 0.86–0.93, REL 0.20–0.25, ASM/HW 0.43–0.46, ASM/CLW 1.35–1.51, CLW/CLL 0.89–1.07 (Suppl. material 1: table S2).

**Figure 1.**
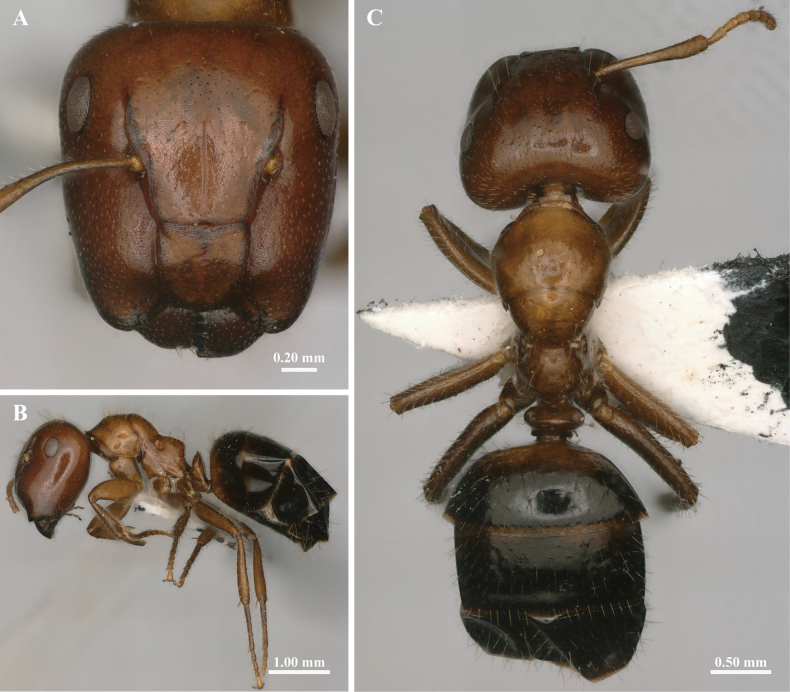
The external morphological characteristics of *Colobopsis minus* major worker. A. Head in full-face view; B. Body in lateral view; C. Body in dorsal view.

In full-face view (Fig. 1A), head phragmotic, occipital margin nearly straight. The head is weakly truncated, the truncated portion incorporating the lower part of the clypeus, genae and the upper surface of the mandibles. Clypeus elongate-rectangular (CLW/CLL ranges from 0.89 to 1.07), the anterolateral extremities separated from the clypeus by a well-marked sulcus. Frontal carinae relatively long; frontal lobes absent. Antennal carinae exposed. There is a distinctive groove in the center of the frontal region, reaching to a position just below middle of eyes. Scape projecting less than 1/5 of its length beyond the occipital border.

In lateral view (Fig. 1B), compound eyes smaller (REL 0.20–0.25; REL of minor worker approx. 0.34), located behind the midline of the head, and all worker castes lack ocelli. Mesonotum strongly sloped backward; meso-propodeal suture very concave. Metanotal spiracles situated at dorsal surface of the propodeum. Mesosoma raised behind the meso-propodeal suture. Base of propodeum inclined forward, declivity concave, forming an almost obtuse right angle with the base. Petiole thin, scale shape, convex anteriorly and flat posteriorly in lateral view.

In dorsal view (Fig. 1C), pronotum broadly rounded, posterior margin enclosing half of mesonotum. Promesonotal suture is a transverse impression. Apex of petiole slightly concave. Gaster oval.

Body shiny, head and mesosoma with very faint reticular. The whole body (including scapes and legs) furnished with abundant yellowish white long hairs, except for clypeus and frontal area, where the hairs sparse. Head, funiculus, mesosoma, petiole and base of the first segment of gaster brownish red. Rest of gaster black. Scape and legs brown.

***Minor worker*** (*n* = 6): TL 3.75–5.06, HL 0.94–1.07, HW 0.83–0.97, ASM 0.38–0.42, CLL 0.26–0.31, CLW 0.43–0.49, SL 0.83–0.94, EL 0.29–0.32, CI 0.84–0.91, SI 0.97–1.02, PW 0.63–0.69, REL 0.33–0.35, ASM/HW 0.43–0.47, ASM/CLW 0.83–0.88, CLW/CLL 1.58–1.65.

**Figure 2.**
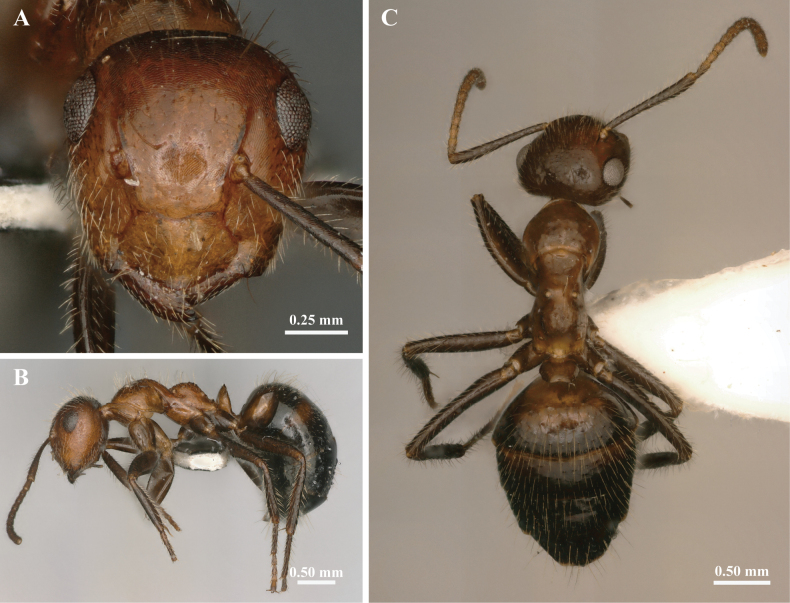
The external morphological characteristics of *Colobopsis minus* minor worker. A. Head in full-face view; B. Body in lateral view; C. Body in dorsal view.

In full-face view (Fig. 2A), head slightly longer than broad; occipital border slightly convex, and the occipital corner broadly rounded. Clypeus wider than long, with the anterior margin rounded. The anterolateral extremities of clypeus are distinguished from the rest of clypeus by an impression extending from the anterior tentorial pit to the clypeal margin, where the suture between the clypeus and malar is weak. Mandibles narrow, masticatory margin with five teeth (Suppl. material 2: fig. S2A). Antennae scape projecting beyond the occipital border ~2/5 of its length.

In lateral view (Fig. 2B), head nearly oval, the connection between frontal carina and clypeus is smooth, with no obvious truncation.

In dorsal view (Fig. 2C), mesonotum distinctly narrower than pronotum. Metanotal spiracles closer to dorsal surfaces than major worker.

The whole body (including antennae and leg) furnished with abundant yellowish white long hairs. The rest of the features are the same as those of the major worker.

**Figure 3.**
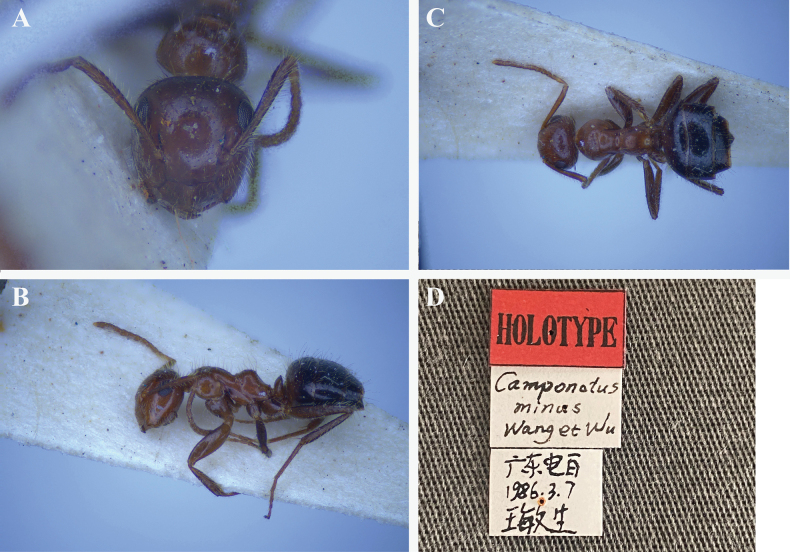
Holotype worker of *Colobopsis minus*. A. Head in full face view; B. Body in lateral view; C. Dorsal view; D. Label of the holotype. (Photo provided by Natural History Museum of the Chinese Academy of Forestry)

***Notes*.** The holotype of *Camponotus minus* was (minor worker) collected by Minsheng Wang in Dianbai County, Guangdong Province, China, in 1986. It is deposited in the collection of the Natural History Museum of the Chinese Academy of Forestry. The holotype is relatively dark in color, has fewer brownish red lumps at the base of the first gaster tergite, and exhibits a more pronounced mesonotal protuberance (Fig. 3). Apart from the above characteristics, all other features are similar to those of the specimen examined in this study, and the species can be identified as conspecific. In full-face view, frontal carinae are relatively short, widely separated (distance approximately half of HW), and the antennal insertions are also relatively well separated, located at approximately the mid-length of the frontal carinae. Key diagnostic indices (ASM/HW and ASM/CLW) fall within the range documented for *Colobopsis* (ASM/HW 0.31–0.47; ASM/CLW 0.60–0.98) (Ward et al. 2016).

***Gyne*** (*n* = 6): TL 8.90–9.85, HL 1.67–1.76, HW 1.55–1.58, ASM 0.64–0.70, CLL 0.51–0.57, CLW 0.47–0.53, SL 1.07–1.15, EL 0.51–0.57, CI 0.90–0.94, SI 0.68–0.73, PW 1.40–1.58, REL 0.33–0.36, ASM/HW 0.41–0.44, ASM/CLW 1.30–1.49, CLW/CLL 0.84–1.04.

**Figure 4.**
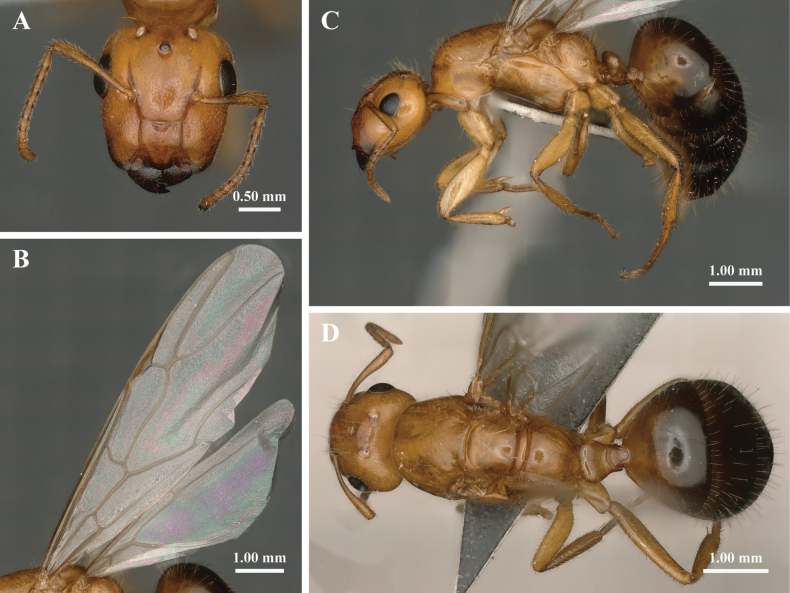
The external morphological characteristics of *Colobopsis minus* gyne. A. Head in full-face view; B. Lateral view of forewings and hindwings; C. Body in lateral view; D. Dorsal view.

In full-face view (Fig. 4A), head resembles that of the major worker but is more elongated (CI 0.90–0.94; CI of all major workers measured are greater than 0.94). Three ocelli present. Compound eyes larger than those of the major worker, situated laterally beyond midlength of the head. Clypeus elongate-rectangular, with its length slightly longer than width.

In lateral view (Fig. 4B, C), petiole similar to that of the major workers, but appearing lower and thicker. Wings whitish hyaline, with pale yellow veins and stigma. Forewings lacking subMarginal 2 and Discoidal cell, Media 4 vein extends to the outer margin, corresponding to the “formica type” in typology III (Cantone 2018). For hindwings, basal and subbasal cells are always present, but the Media 2 vein and Jugal lobe are absent, classifying them as typology II (Fig. 4B).

In dorsal view (Fig. 4D), pronotum nearly trapezoidal, with other characteristics similar to major worker.

Body smooth and yellowish brown; petiole and basal portion of the first gastral tergite slightly darker; mandibles and remaining gaster black. Whole body, including antennae and legs, covered with abundant yellowish-white long hairs. Other morphological features similar to those of the major worker in general appearance.

**Male** (*n* = 6): TL 4.90–5.30, HL 0.76–0.88, HW 0.77–0.82, ASM 0.19–0.24, CLL 0.20–0.26, CLW 0.32–0.36, EL 0.31–0.33, CI 0.92–1.04, PW 0.89–0.93, REL 0.38–0.42, ASM/HW 0.24–0.30, ASM/CLW 0.56–0.71, CLW/CLL 1.38–1.62.

**Figure 5.**
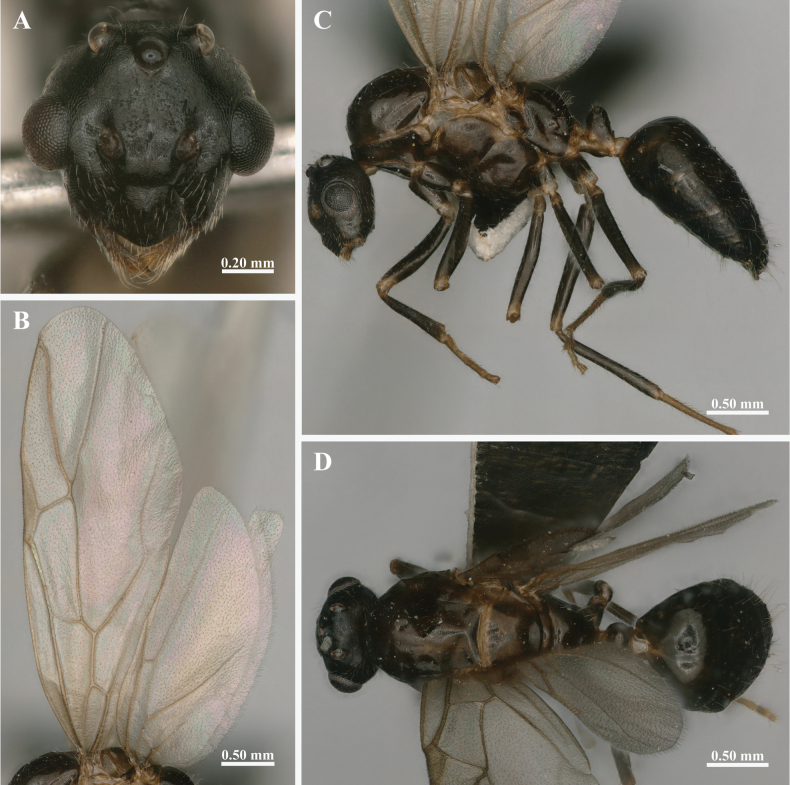
The external morphological characteristics of *Colobopsis minus* male. A. Head in full-face view; B. Lateral view of forewings and hindwings; C. Body in lateral view; D. Dorsal view.

In full-face view (Fig. 5A), head oval, slightly longer than broad including the mandibles, with prominent compound eyes and three well-developed ocelli. Compound eyes located along the lateral midline of head; ocelli large, situated near the occipital margin. Genae converge slightly anteriorly; posterior corners broadly rounded. Clypeus sharply keeled, slightly wider than long, with sides diverging moderately towards the anterior margin, the anterolateral extremities separated from the clypeus by a well-marked sulcus and appearing to form an independent triangular region.

In lateral view (Fig. 5B, C), wings similar to those of the gyne, with the forewings are categorized as “formica type” in Typology III due to the absence of subMarginal 2 and Discoidal cell, Media 4 vein extending to the outer margin; hindwings are categorized as Typology II for the absence of the Media 2 vein and Jugal lobe (Cantone 2017).

In dorsal view (Fig. 5D), similar to major worker.

Slightly larger than minor worker. Body smooth, predominantly black, with the mesosoma slightly lighter. Mandibles, tarsi, and articulation of all body parts (including wing bases and legs) sordid yellow. Hairs whitish, suberect, much scattered, cover densely on the head, legs, and gaster.

### ﻿Biology

**Figure 6.**
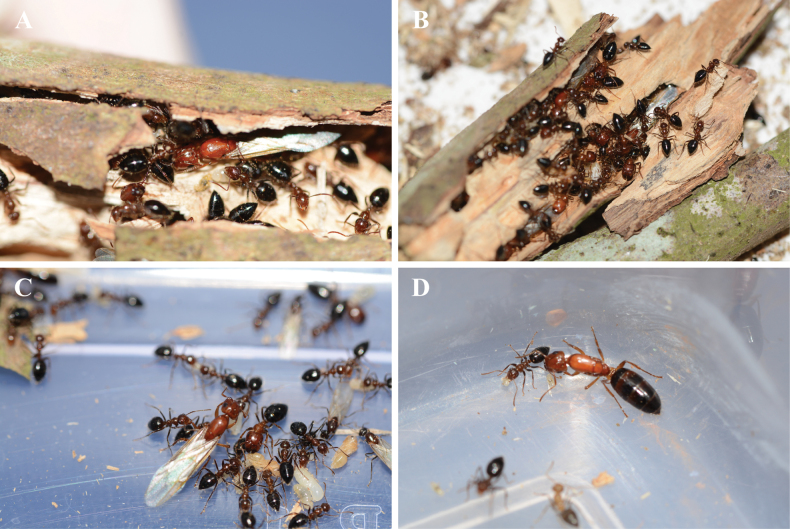
Ecological features of *Colobopsis minus*. A, B. Nest cavities of *C. minus* inside twigs of the host tree; C. Larvae, naked pupae and winged reproductives tended by workers; D. Dealate queen of *C. minus*.

*Colobopsis minus* is strictly arboreal, nesting in galleries within dead, hollow branches, and twigs of the host trees (Fig. 6A, B). The colonies collected in this study were found inhabiting in dead bamboo cavities (colony GDGQ) and branches of cf. *Cinnamomum burmanni* (colony GDTQ). Similar to other *Colobopsis* species, the pupae of *C. minus* are naked (Fig. 6B, C), which distinguishes them from those of *Camponotus*, whose pupae are always enclosed in cocoons (Wheeler 1904).

### ﻿Phylogenetic analysis

**Figure 7.**
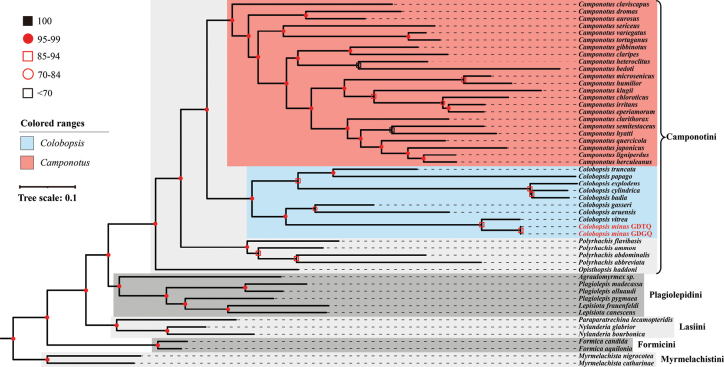
Maximum likelihood (ML) phylogenetic tree of the ant subfamily Formicinae based on COI sequences, with *Myrmelachista* used as the outgroup. The tree resolves five major clades, corresponding to the tribes Camponotini, Plagiolepidini, Formicini, Lasiini, and Myrmelachistini.

The maximum likelihood phylogenetic analysis revealed that the sampled species within the tribe Camponotini are divided into four distinct clades: *Camponotus*, *Colobopsis*, *Polyrhachis*, and *Opisthopsis* (Fig. 7). *Colobopsis minus* is robustly nested within the *Colobopsis* clade, with strong nodal support distinguishing it from the *Camponotus* lineage. This placement is further supported by notable genetic divergence, as indicated by the *p*-distances calculated in MEGA (Suppl. material 1: table S4). The closest relative of *C. minus* is *C. vitrea* (*p*-distances = 0.0882), which is consistent with their phylogenetic relationship and morphological similarity (Fig. 7, Suppl. material 2: fig. S3). However, due to the limitation of a single mitochondrial gene, the deeper parts of the COI tree need further investigation.

### ﻿Keys to *Colobopsis* species of China

In addition to *C. minus*, nine other species of the genus *Colobopsis* have been recorded from China according to AntMaps (2025): *C. badia*, *C. ceylonica*, *C. cotesii*, *C. laotsei*, *C. leonardi*, *C. nipponica*, *C. rothneyi*, *C. taivanae*, and *C. vitrea*. The following keys are constructed based on the morphological characteristics of the major workers and minor workers of *Colobopsis* species known from China and with reference to the key in Dhadwal and Bharti (2024).

#### ﻿Key to species recorded in China based on major worker

**Table:** 

1	Presence of pubescence on the body surface	**2**
–	Absence of pubescence on the body surface	**3**
2	Body shining, with only pubescence on head, gaster, and tips of femur	***C. laotsei***
–	Body sculpture covered by a dense layer of sericeous pubescence	***C. leonardi***
3	Meso-propodeal suture inconspicuous, with only a shallow transverse impression	**4**
–	Meso-propodeal suture distinct, concave downwards into a slope	**7**
4	In lateral view, head weakly truncate; metanotum raised; petiole squaliform and thin	**5**
–	In lateral view, head strongly truncate; metanotum flatted; petiole in form of globular node, shorter and lower	**6**
5	Widely emarginate at promesonotal suture; in profile petiole decidedly thinner, with sharper superior border	***C. ceylonica***
–	Promesonotal suture less concave; in profile petiole thick, transverse above	***C. nipponica***
6	Body yellowish-brown, abdomen darker; mandible reticulate wrinkled, without punctures	***C. rothneyi***
–	Body blackish brown, with yellowish edges on the abdominal segments; mandible smooth and shiny, punctures fine	***C. taivanae***
7	Sides of propodeum smooth and shiny	**8**
–	Sides of propodeum rugulose or reticulate-punctate	**9**
8	Dark brown, limbs and funiculus lighter colored, gaster darker; mandible, clypeus, the lower part of the frontal area and cheek reddish-brown	***C. vitrea***
–	Head, funiculus, mesosoma, petiole and base of the first segment of gaster brownish red. Rest of gaster black. Scape and legs brown	***Colobopsis minus* (Wang & Wu, 1994), comb. nov.**
9	In full face view, head strongly truncate; metanotum raised above and conical	***C. cotesii***
–	In full face view, head oval, weakly truncate; metanotum obliquely truncate	***C. badia***

#### ﻿Key to species recorded in China based on minor worker

**Table:** 

1	Presence of pubescence on the body surface	**2**
–	Absence of pubescence on the body surface	**3**
2	Body shining, with only pubescence on head, gaster, and tips of femur	***C. laotsei***
–	Body sculpture covered by a dense layer of sericeous pubescence	***C. leonardi***
3	Meso-propodeal suture inconspicuous, with only a shallow transverse impression	**4**
–	Meso-propodeal suture distinct, concave downwards into a slope	**7**
4	Petiole squaliform and thin	**5**
–	Petiole in form of globular node, shorter and lower	**6**
5	Pale testaceous yellow, tarsi and abdomen darker, with yellowish-brown edges on abdominal segments; metanotum flatted	***C. ceylonica***
–	Overall dark brown, appendages paler and the gaster immaculate; metanotum raised	***C. nipponica***
6	Body yellowish-brown, abdomen darker; mandible reticulate wrinkled, without punctures	***C. rothneyi***
–	Body blackish brown, with yellowish edges on the abdominal segments; mandible smooth and shiny, punctures fine	***C. taivanae***
7	Sides of propodeum smooth and shiny	**8**
–	Sides of propodeum rugulose or reticulate-punctate	**9**
8	Body completely black; Scape longer (SL = 1.33–1.43)	***C. vitrea***
–	Head, funiculus, mesosoma, petiole and base of the first segment of gaster brownish red. Rest of gaster black. Scape and legs brown; Scape shorter (SL = 0.83–0.94)	***Colobopsis minus* (Wang & Wu, 1994), comb. nov.**
9	Widely emarginate at the meso-metanotal suture; metanotum raised above, rounded or conical; abdomen black, with two spots at its base and two lateral spots at the base of the 3^rd^ segment testaceous yellow	***C. cotesii***
–	The degree of concavity of the meso-metanotal suture; metanotum obliquely truncate; abdomen reddish brown, without any spots	***C. badia***

## ﻿Discussion

Our study validates the reclassification of *Colobopsis minus* (Wang & Wu, 1994), comb. nov., which was previously misplaced within *Camponotus*. By examining all castes, including minor and major workers, gynes and males, we provide detailed morphometric data, high-resolution images, and molecular evidence that conclusively align this species within *Colobopsis*. Notably, the presence of a phragmotic major worker (though variable across species) and the absence of cocoon-spinning in the larval stage represent features commonly associated with *Colobopsis*.

The transfer of *Camponotus minus* to *Colobopsis*, as strongly supported by our morphological and COI data, is definitively confirmed by a recent ant phylogenomic study (Vizueta et al. 2025). Notably, this study sequenced whole genomes from a vast diversity of ants, including specimens of *Colobopsis minus* originating from the same colony (GDTQ) utilized in our molecular work. However, the deeper phylogenetic structure of our COI tree, which suggests a sister-group relationship between *Colobopsis* and *Camponotus*, is inconsistent with more comprehensive phylogenomic studies (Blaimer et al. 2015; Vizueta et al. 2025; Ward et al. 2025), which places *Colobopsis* outside a clade containing *Camponotus* and *Polyrhachis*); this discrepancy underscores the known limitation of a single mitochondrial gene for resolving ancient phylogenetic splits. Nevertheless, both studies conclusively agree on the taxonomic conclusion of this study, that *Colobopsis minus* (Wang & Wu, 1994), comb. nov. is a valid species within the genus *Colobopsis*.

This study also highlights the broader challenges in ant taxonomy. Similar to the original description of *C. minus*, many early taxonomic publications simply listed type specimen repositories without providing accessible morphological details or caste-specific descriptions (e.g., Chang and He 2002a, 2002b). Such practices complicate subsequent identification efforts, requiring direct examination of type specimens and thus increasing logistical, temporal, and financial costs. This barrier is particularly prominent in genera such as *Cataglyphis* and *Messor* (Chang and He 2002c; Song and He 2009) where descriptions are often based on limited type series and accompanied by brief diagnoses or on limited type material, brief textual diagnoses, and/or rudimentary illustrations. *Colobopsis minus* stands as another typical case lacking critical diagnostic characters and caste coverage in its original account. These shortcomings hinder biodiversity assessments, phylogenetic inference, and conservation prioritization. Recently, as one of the main storage institutions for type specimens of Chinese ant species, Southwest Forestry University published the book ‘Type Specimens of Ants Housed in Southwest Forestry University’ (Xu 2024), which represents progress toward closing these gaps by documenting 136 type specimens across 39 genera. However, the issue persists, as a substantial proportion of Chinese ant species still lack comprehensive morphological descriptions and/or high-resolution type specimen images, with available data frequently limited to a single caste.

Advances in integrative taxonomy now offer solutions. Molecular phylogenetics, particularly approaches using ultra-conserved elements (UCEs), mitogenomes, or barcoding sequences such as COI and COII, can complement traditional morphology-based identification and provide a stable framework for resolving taxonomic relationships at multiple scales (Brady et al. 2006; Moreau et al. 2006; Branstetter et al. 2017; Starr 2021). Our study applies these principles by: (1) re-examining holotypes to validate original diagnoses; (2) supplementing all caste descriptions with morphometric data and high-resolution images; (3) sequencing COI barcode, with deposition of barcode sequences in public databases to enable reproducible, large-scale identification; and (4) integrating phylogenetic data to evaluate genus-level placement. This workflow could standardize revisions for problematic taxa which exhibit unresolved species boundaries due to limited morphological information.

Ultimately, resolving taxonomic uncertainties in hyperdiverse groups such as ants requires coordinated efforts. While online platforms like AntWeb (2025), AntWiki (2025), and AntCat (Bolton 2025) have greatly improved data accessibility, regional collections (e.g., SWFU, GXNU) should continue to prioritize the digitization of type specimens and publishing standardized data-rich species descriptions. By adopting reproducible, integrative frameworks such as those advocated by Padial et al. (2010), the myrmecological community can overcome historical limitations and build a more robust foundation for downstream research in systematics, ecology, biogeography, and invasive species management.

## Supplementary Material

XML Treatment for Colobopsis minus

## ﻿Additional information

### Use of AI

No use of AI was reported.

### Author ORCIDs

Hao Ran https://orcid.org/0000-0001-5462-3615

Qionghua Gao https://orcid.org/0000-0003-2365-1431

### Data availability

All of the data that support the findings of this study are available in the main text or Supplementary Information.

## Supplementary materials

Supplementary material 1 Supplementary information 1 Data type xlsx https://binary.pensoft.net/file/1466211This dataset is made available under the Open Database License (http://opendatacommons.org/licenses/odbl/1.0/). The Open Database License (ODbL) is a license agreement intended to allow users to freely share, modify, and use this Dataset while maintaining this same freedom for others, provided that the original source and author(s) are credited.Yonghao Ye, Hao Ran, Qionghua Gao

Supplementary material 2 Supplementary information 2 Data type zip Explanation note **figure S1.** The lateral view of head of minor worker of *Colobopsis* and *Camponotus*. **figure S2.** The full-face view of head of minor worker of *Colobopsis* and *Camponotus*. **figure S3.** The lateral view of *Colobopsis* major worker and *Camponotus* soldier. https://binary.pensoft.net/file/1466212This dataset is made available under the Open Database License (http://opendatacommons.org/licenses/odbl/1.0/). The Open Database License (ODbL) is a license agreement intended to allow users to freely share, modify, and use this Dataset while maintaining this same freedom for others, provided that the original source and author(s) are credited.Yonghao Ye, Hao Ran, Qionghua Gao
