# Supplementary material for: ﻿A taxonomic review of Colobopsis minus (Wang & Wu, 1994), comb. nov. from China, with description of all castes (Hymenoptera, Formicidae)
Source: Zookeys. 2025 Nov 14;1260:75–91. doi: 10.3897/zookeys.1260.166957 (PMC12639361; doi:10.3897/zookeys.1260.166957)
Supplement: Supplementary material 2 — Supplementary information 2 [file zookeys-1260-075_article-166957__-s002.zip › 166957_0R-1-A_supplementary_figure_legends.docx]

**Supplementary figure legends**

**Figure S1.** The lateral view of head of minor worker of *Colobopsis* and *Camponotus*. **A.** *Colobopsis minus*. **B.** *Colobopsis vitrea* (CASENT0217714). **C.** *Camponotus aurosus* (CASENT0064815). **D.** *Camponotus dromas* (CASENT0280174). Images from AntWeb (www.antweb.org); photographers: Michele Esposito (B), April Nobile (C) and Will Ericson (D).

**Figure S2.** The full-face view of head of minor worker of *Colobopsis* and *Camponotus*. **A.** *Colobopsis minus*. **B.** *Colobopsis vitrea* (CASENT0217714). **C.** *Camponotus aurosus* (CASENT0064815). **D.** *Camponotus dromas* (CASENT0280174). Images from AntWeb (www.antweb.org); photographers: Michele Esposito (B), April Nobile (C) and Will Ericson (D).

**Figure S3.** The lateral view of *Colobopsis* major worker and *Camponotus* soldier. **A.** *Colobopsis minus*. **B.** *Colobopsis vitrea* (CASENT0280181). **C.** *Camponotus aurosus* (CASENT0135247). **D.** *Camponotus dromas* (JDM32-001165). Images from AntWeb (www.antweb.org); photographers: Will Ericson (B), April Nobile (C) and Brian Heterick (D).
