# Supplementary figures and images for: ﻿A taxonomic review of Colobopsis minus (Wang & Wu, 1994), comb. nov. from China, with description of all castes (Hymenoptera, Formicidae)
Source: Zookeys. 2025 Nov 14;1260:75–91. doi: 10.3897/zookeys.1260.166957 (PMC12639361; doi:10.3897/zookeys.1260.166957)

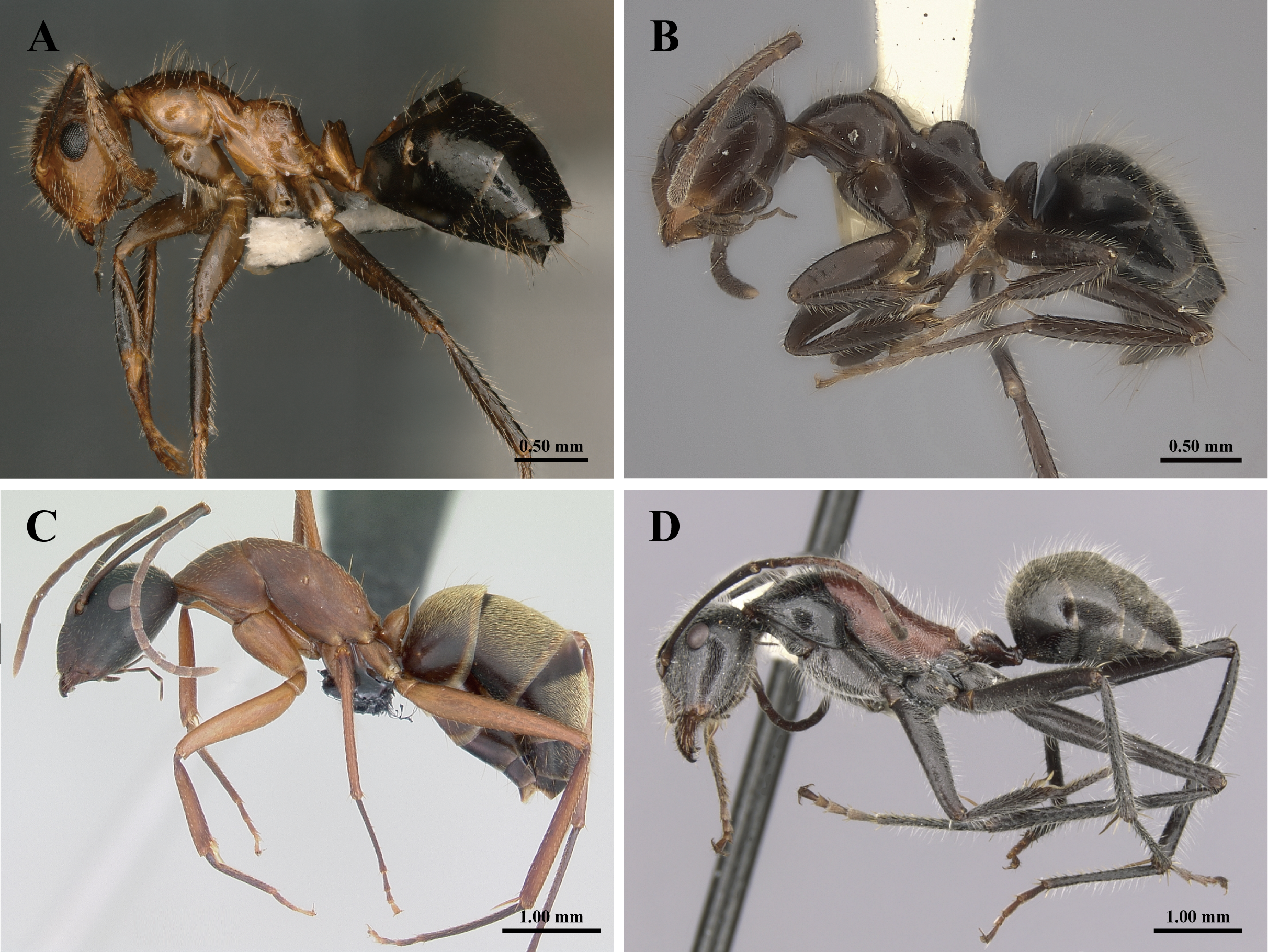

Supplement: Supplementary material 2 — Supplementary information 2 [file zookeys-1260-075_article-166957__-s002.zip › 166957_0R-1-A_Figure_S1_The_lateral_view_of_head_of_minor_worker_of_Colobopsis_and_Camponotus.tif]

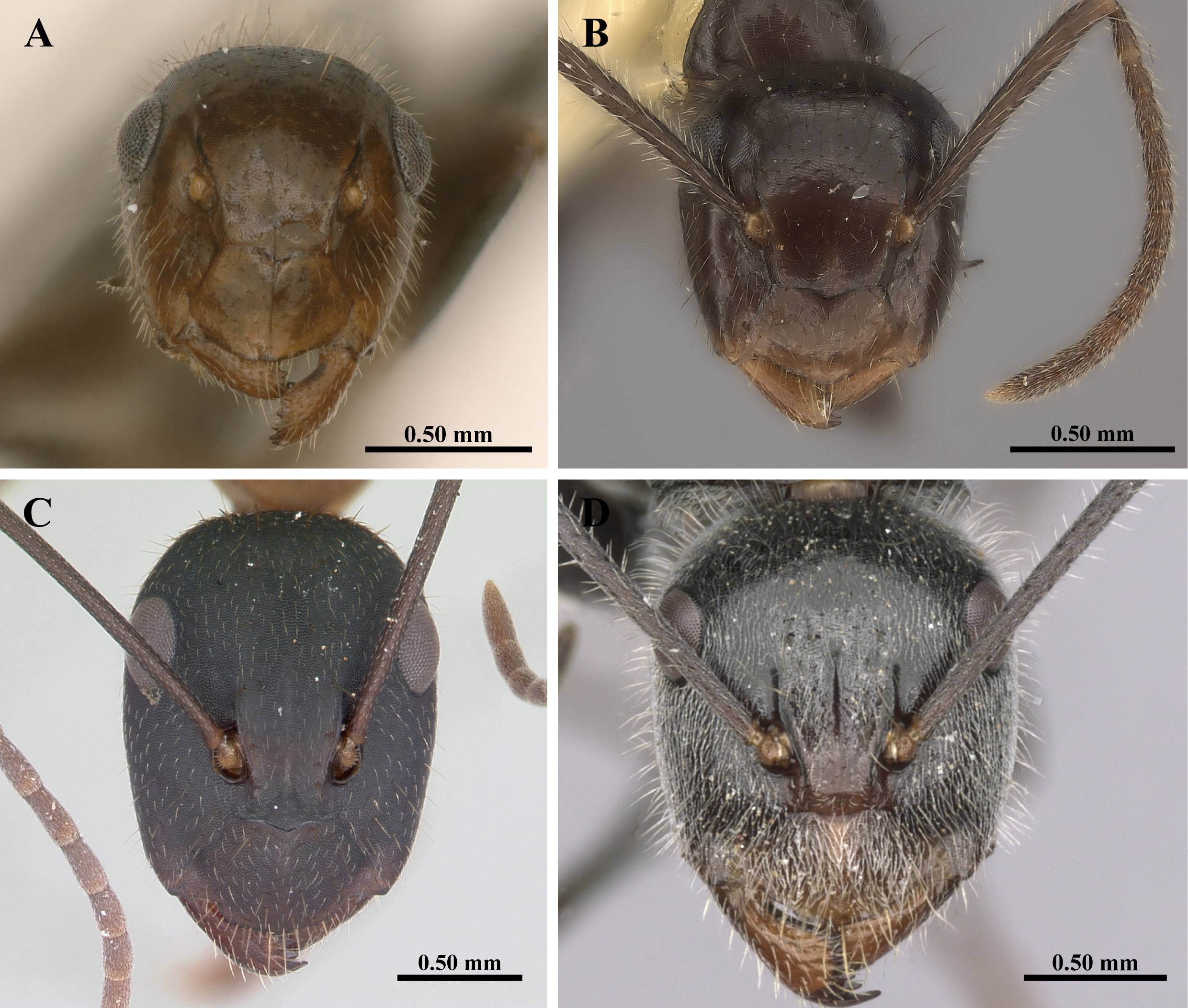

Supplement: Supplementary material 2 — Supplementary information 2 [file zookeys-1260-075_article-166957__-s002.zip › 166957_0R-1-A_Figure_S2_The_full-face_view_of_head_of_minor_worker_of_Colobopsis_and_Camponotus.tif]

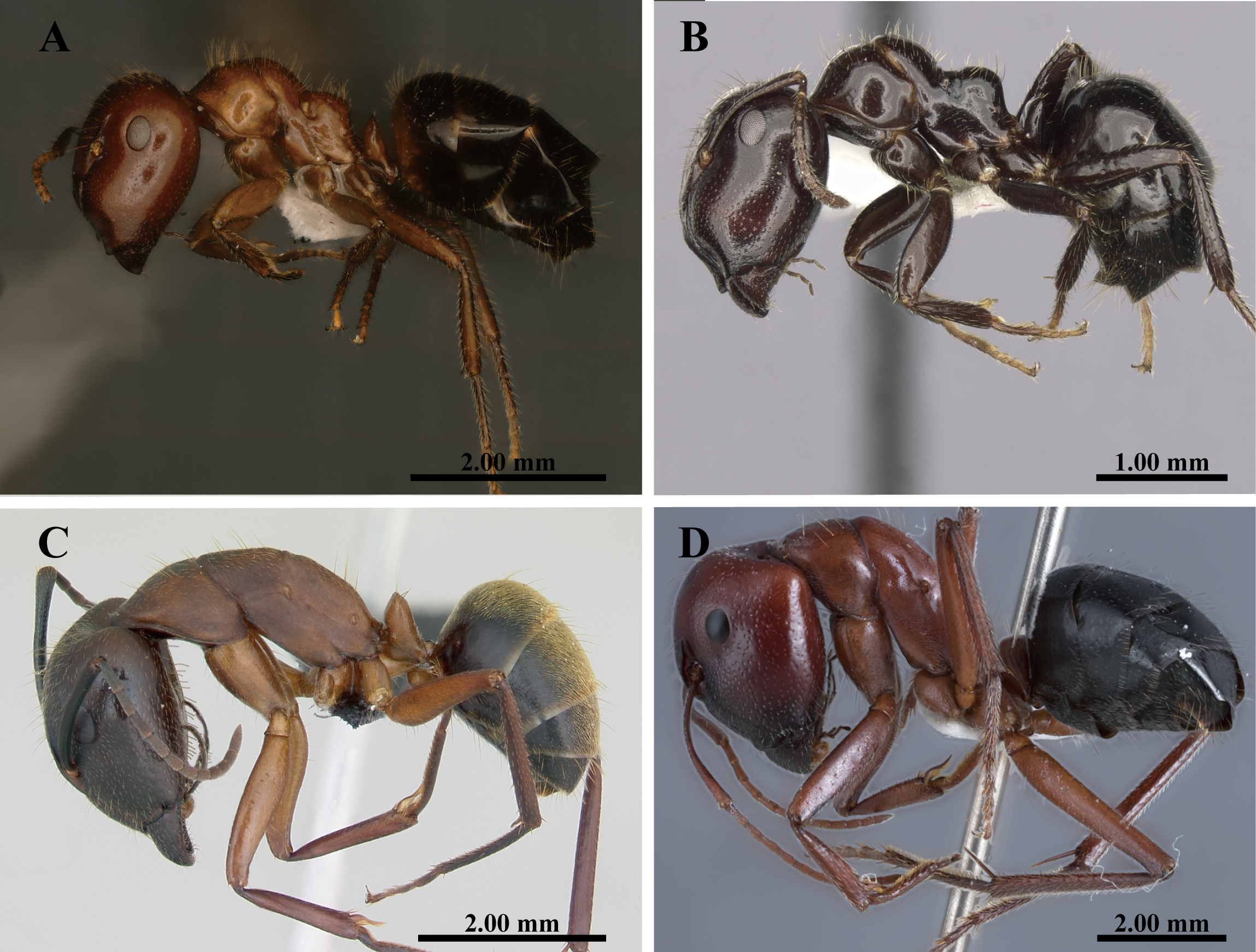

Supplement: Supplementary material 2 — Supplementary information 2 [file zookeys-1260-075_article-166957__-s002.zip › 166957_0R-1-A_Figure_S3_The_lateral_view_of_Colobopsis_major_worker_and_Camponotus_soldier.tif]
